# Transcriptomic Analysis Reveals the Correlation between End-of-Day Far Red Light and Chilling Stress in *Setaria viridis*

**DOI:** 10.3390/genes13091565

**Published:** 2022-08-31

**Authors:** Shilei Sun, Qingjia Liu, Xiuru Dai, Xianglan Wang

**Affiliations:** State Key Laboratory of Crop Biology, College of Agronomic Sciences, Shandong Agricultural University, Tai’an 271018, China

**Keywords:** *Setaria viridis*, EOD-FR, chilling, transcriptional dynamics, WGCNA, *BBX2*

## Abstract

Low temperature and end-of-day far-red (EOD-FR) light signaling are two key factors limiting plant production and geographical location worldwide. However, the transcriptional dynamics of EOD-FR light conditions during chilling stress remain poorly understood. Here, we performed a comparative RNA-Seq-based approach to identify differentially expressed genes (DEGs) related to EOD-FR and chilling stress in *Setaria viridis*. A total of 7911, 324, and 13431 DEGs that responded to low temperature, EOD-FR and these two stresses were detected, respectively. Further DEGs analysis revealed that EOD-FR may enhance cold tolerance in plants by regulating the expression of genes related to cold tolerance. The result of weighted gene coexpression network analysis (WGCNA) using 13431 nonredundant DEGs exhibited 15 different gene network modules. Interestingly, a CO-like transcription factor named *BBX2* was highly expressed under EOD-FR or chilling conditions. Furthermore, we could detect more expression levels when EOD-FR and chilling stress co-existed. Our dataset provides a valuable resource for the regulatory network involved in EOD-FR signaling and chilling tolerance in C_4_ plants.

## 1. Introduction

Abiotic stresses, such as light and temperature stress, affect the growth, productivity, and quality of plants. Because of these environmental challenges, plants must be able to accurately detect slight changes and transmit stress signals within cells and tissues to adjust physiological and biochemical processes.

Chilling is one of the most detrimental abiotic stresses for the plant life cycle. Chilling stress results in a loss of membrane integrity, increased ion efflux, and disorganized cellular metabolism [1]. Due to their rapid adaptation to low temperatures, plants have evolved complex and diverse mechanisms to protect themselves. The response to chilling stress involves numerous genes, such as transcription factors, enzymes, and phytohormone metabolism. In a previous study, the ICE1-CBF-COR signaling pathway was shown to play an important role in plant cold stress [2,3]. C-repeat dehydration responsive element binding (DREB) factors (CBFs), as key transcription factors, activate the expression level of cold-regulated (*COR*) genes that function in cold acclimation [2,4,5,6]. In *Arabidopsis thaliana*, three CBF family members, *CBF1*, *CBF2*, and *CBF3*, are involved in cold acclimation mechanisms when the plant is exposed to nonfreezing temperatures. Transgenic lines with enhanced *CBF1*/*CBF3* expression exhibit improved cold tolerance in plants, consistent with the reduced cold tolerance in loss-of-function mutants [6,7]. However, *CBF2,* acting as a negative regulator, inhibited the expression levels of *CBF1* and *CBF3* to confer enhanced cold tolerance to *cbf2* plants [8]. Transcriptome analysis indicated that 12% of cold-responsive genes are controlled by CBFs [9]. Therefore, some CBF-independent components must function in cold signaling. Among CBF-independent cold signaling pathways, the abscisic acid (ABA)-dependent cold signaling pathway has been studied in depth in recent years. Based on RNA-sequencing data, approximately 10% of ABA-related genes also responded to cold stress [10]. The promoters of *COR* genes, such as *RD29A*, *RD22*, *COR15A*, and *COR47*, containing both C-repeat/dehydration responsive element (CRT/DRE) and ABA response cis-elements (ABREs), also indicate that crosstalk between cold and ABA signaling exists authentically [11].

When plants are grown for yield, light and temperature are the key factors working synergistically to influence the growth state of plants. This evidence suggests that light signaling may be involved in cold-induced gene expression through C/DRE elements in *Arabidopsis thaliana* [12]. Based on previous studies, the expression levels of *CBFs* are regulated by light signaling, such as light quality, circadian rhythm or photoperiod, to promote cold tolerance [13,14,15,16]. Phytochrome B (PHYB) acts as a thermosensor to negatively regulate cold tolerance in *Arabidopsis*, rice, and tomato [17,18,19,20]. A gene named *OsPIL16*, which belongs to the phytochrome interacting factor (PIF) gene family, positively regulates *OsDREB1B* expression. Intriguingly, both in *OsPIL16*-OX lines and *phytochrome B* mutants, the improved expression level of *OsDREB1s* and induced cold tolerance can be detected in rice. Thus, this may be the probable cross-talk between phytochrome B and cold tolerance [21]. The flavin reoxidation rate of Crys from the active state to the inactive state is lower at 15 °C than at 25 °C, suggesting that the concentrations of the active redox forms of Crys could be increased at low temperatures [22]. Moreover, *PIFs* were reported to participate in multiple biological processes in plants, such as fruit color formation, hypocotyl-specific growth, leaf senescence, and circadian oscillator [23,24,25,26,27]. Researchers found that *PIF8* positively regulates cold tolerance by modulating superoxide dismutase (SOD) levels in *Citrus sinensis* [28]. The low ratio of red to far red induces *SlPIF4* accumulation under cold stress, which activates the expression of *SlCBFs* and positively regulates cold tolerance in tomato [29]. *PIF3* negatively regulates the cold acclimation of plants by binding to the promoter of *CBFs* [30]. In addition, acting as the target genes of *HY5*, *BBX7*, and *BBX8* positively regulates the expression level of CBF-independent *COR* genes in response to freezing tolerance [31]. *CCA1* and *LHY*, which are core components of the circadian clock, directly target the promoters of *CBF1-3* [32]. Acting as regulators of circadian clock feedback loops, GI, ZTL, and EC affect the expression of *CBF3* through the *LHY/CCA1* pathway [33]. Under red/far-red ratio conditions, the increased expression of *CBF1-3* has been detected in a manner dependent on the circadian clock [17].

Little is known about crosstalk between light signaling and cold signaling in *Setaria viridis*. In our research, RNA-Seq data were analyzed to evaluate the effects of low temperature and EOD-FR signaling in seedlings of *Setaria viridis*. These time series data are excellent materials for detecting transcriptional dynamics, for revealing which genes are up- or downregulated during the different handling times with 4 °C or EOD-FR, and particularly for inferring transcription factors strongly induced by these two stresses. Functional category enrichment was performed to analyze the biological processes that DEGs enriched under three stress conditions. Subsequently, we used WGCNA to construct gene co-expression network that captured modules with 4 °C and EOD-FR specificity. A total of 15 gene modules were identified from this network, highlighting functions in stress responses, metabolism, signaling, and nutrients pathways. As examples, we describe in detail the brown module involved in abiotic stress. In particular, these modules contain transcription factors that act as potential gene expression regulator for the genes within the same modules. According to the analysis, a candidate gene named *BBX2* was selected to study gene function, demonstrating the usefulness of this network.

## 2. Materials and Methods

### 2.1. Plant Material for RNA-Seq Experiments

**Figure 1:**
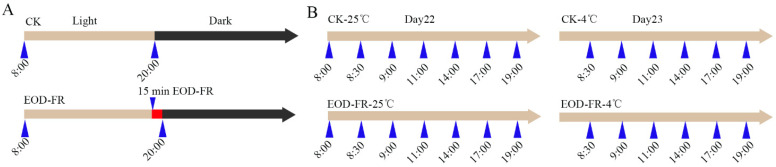
A schematic representation of the light and cold treatments. (**A**) The growing environment of plants before sampling. The CK group grew under normal conditions (light: 12 h; dark: 12 h; temperature: 25 °C). EOD-FR group was similar to the CK group except that it was given Far-red light for 15 min after the light ended. After 21 days in this environment, the plants of the aerial parts were sampled. (**B**) Setting of sampling time. On day 22, we set up a total of 7 sampling points. On day 23, we moved the plants to 4 °C at 8 a.m. and sampled plants at the 6 time points.

To correlate EOD-FR with cold stress, the growth conditions of *Setaria viridis* are set as follows. Briefly, the control group plants were grown in a light-emitting diode growth chamber at 12 h light/12 h dark cycles at 24 h under the White light. While, the experimental group plants were grown under White light (WL) plus 15 min End-of-day (EOD) Far-red. Three weeks later, samples were harvested as the sampling point shown in Figure 1.

### 2.2. Total RNA Isolation

Total RNA was extracted from seedlings above ground as previously described [34]. The extracted RNA was used to construct RNA-Seq library.

### 2.3. RNA-Seq Library Construction, Sequencing and Analysis

The raw reads were trimmed with Trimmomatic [35] with parameter ‘LEADING:3 TRAILING:3 SLIDINGWINDOW:4:15′. Filtered reads were mapped to reference genome Setaria viridis as available at Phytozome using Tophat v2.1.1 [36] with parameter ‘-N 1‘. FPKM (fragments per kilobase per million reads) of each treatment was derived by Cufflinks [37]. DESeq2 [38] was applied to detect differentially expressed genes (DEG) between control and treatment conditions. An FDR ≤ 0.05 and the absolute value of the log_2_ (fold change assessed by FPKM values) ≥ 1 were used as the threshold to determine significance. The FPKM of the gene was used for analysis of PCA.MAPMAN enrichment analysis.

We performed MAPMAN enrichment analysis using R Studio and the clusterProfiler package (clusterProfiler: an R package for comparing biological themes among gene clusters.). To use the enrich MAPMAN function in this package, we used input data consisting of cluster information and the *Setaria viridis* annotation project database ID. In addition, we chose data for organism code and filtered out results by applying adjusted p-value cut-offs of less than 0.01, as in other studies. For visualization of the results, we used TBtools to modify the figure.

### 2.4. Gene Network Construction and Visualization

Co-expression networks were constructed using the WGCNA (v1.29) package in R [39]. The average FPKM values of 13431 genes were imported into WGCNA for constructing unsigned co-expression network. The modules were obtained using the automatic network construction function blockwiseModules with default settings, except that the power is 10, TOM-Type is signed, minModuleSize is 30, and mergeCutHeight is 0.25. The total connectivity and intramodular connectivity (function softConnectivity), kME (for modular membership, also known as eigengene-based connectivity), and kME-P value were calculated for the 10438 genes, which were clustered into 14 modules. The networks were visualized using Cytoscape_v.3.0.0.

## 3. Results

### 3.1. Transcriptome Atlas under EOD-FR and Cold Treatment

We performed an EOD-FR and cold assay to identify transcripts that are differentially expressed (DE) in *Setaria viridis* seedlings in a conventional growth chamber. Plantings were subjected to four different treatments as follows: Control-25 °C (the normal growth condition), Control-4 °C (cold stress), EOD-FR-25 °C (FR treatment), and EOD-FR-4 °C (FR treatment and cold stress). For Control-25 °C, the seedlings were grown under normal conditions. Control-4 °C means the plants were treated with 4 °C cold stress at 8:00 a.m. EOD-FR-25 °C, the plants were treated with 15 min FR light at 20:00 every day. EOD-FR-4 °C, the FR treatment was the same as the EOD-FR-25 °C condition, and then the plants were removed to 4 °C at 8:00 a.m. Samples were collected based on aerial parts of seedlings beginning at 22-day-old plants and finishing at 23-day-old plants (Figure 1). Based on the experimental design, transcriptional dynamics can be identified under EOD-FR and cold stress.

**Figure 2:**
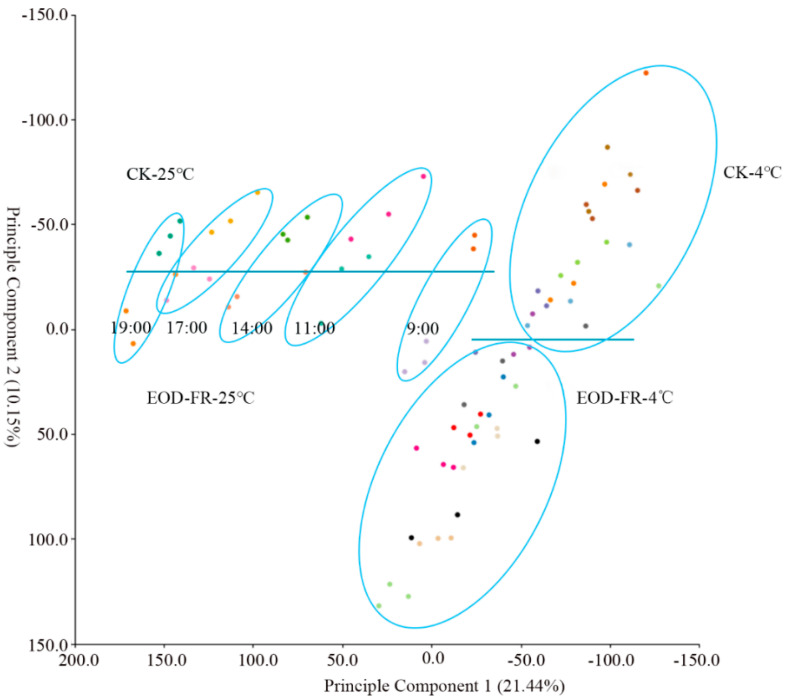
PCA of RNA-Seq data under EOD-FR and cold stress conditions.

To formulate the relationships among the different transcriptomes, principal component analysis (PCA) was executed on the complete RNA-Seq dataset. The first principal component (PC1) and the second principal component (PC2) accounted for 31.59% of the variance. The analyzed results showed that the expression profiles of the different sample collection times were well ordered and separated by PC1 under the Control-25 °C and EOD-FR-25 °C conditions (Figure 2), suggesting similar transcriptional regulation of different seedlings at the same time. However, after cold treatment, the expression profiles at different time points became chaotic and disordered, indicating that the cold treatment had a great impact on gene expression. Additionally, the seedlings handled by EOD-FR were separated from the seedlings grown in white lights by PC2, which may be due to the FR influence.

### 3.2. Functional Category Enrichment under Chilling Stress

**Figure 3:**
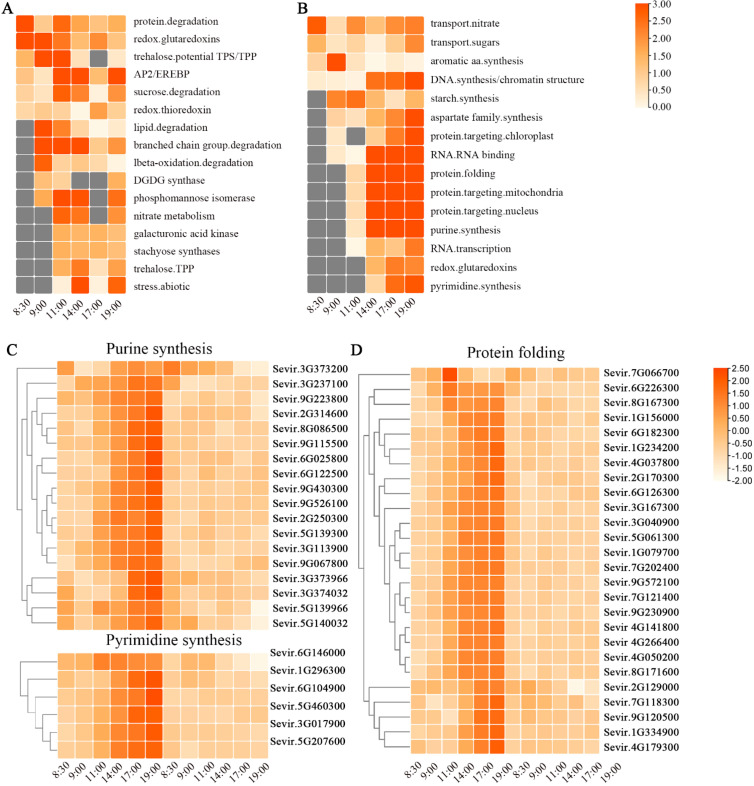
Functional category enrichment among DEGs after cold stress. (**A**) MAPMAN enrichment analysis of upregulated genes after cold stress. Only significant categories (FDR < 0.05) were displayed. (**B**) MAPMAN enrichment analysis of downregulated gene after cold stress. Only significant categories (FDR < 0.05) were displayed. (**C**,**D**) The expression pattern of genes were involved in purine and pyrimidine synthesis (**C**) and proteins folding (**D**) at the six time points.

To characterize the gene response to cold stress in *Setaria viridis*, we compared the expression levels of genes in the Control-4 °C samples with the expression levels of genes in the Control-25 °C samples. Based on the criteria of adjusted *p* value < 0.01 and | log_2_ (fold change) | ≥ 1, 7911 DEGs were observed in all samples (Supplementary Table S1). Furthermore, an increased number of DEGs were detected with the time of the low-temperature treatment, indicating that an increasing number of genes participate in the fight against cold stress (Supplementary Figure S1). To analyze the effects of low-temperature treatment on *Setaria viridis*, we carried out MAPMAN annotation to analyze the functional enrichment of DEGs. At the early stage of low-temperature stress (8:00–11:00), the upregulated genes were concentrated mainly in raffinose synthesis, TPS/TPP signaling, amino acid degradation, lipid degradation, and protein degradation (Figure 3A and Supplementary Table S2). Raffinose, acting as an osmotic regulator, plays an important role in low-temperature stress in plants [40]. TPS/TPP plays a significant role in plant stress tolerance. *OsTPP1*, which encodes a key enzyme for trehalose biosynthesis, contributes most significantly to chilling tolerance in rice [41,42]. This result indicated that the accumulation of soluble sugars, such as raffinose and trehalose, was one of the material bases of chilling tolerance in *Setaria viridis*. In addition, plants began to degrade various nutrients to prepare for resistance to low-temperature stress and to transmit stress information through various signaling pathways. With the extension of cold treatment time (14:00–19:00), carbohydrate and amino acid transport, protein posttranslational modification, lignin synthesis, nitrate nitrogen metabolism, and digalactosyldiacylglycerol (DGDG) synthesis were the most significantly enriched (Figure 3A and Supplementary Table S2). Monogalactosyldiacylglycerol (MGDG)/DGDG, an important component of the chloroplast membrane and thylakoid membrane, is a prominent feature in plant cold tolerance [43]. The changes in channel number, membrane composition, and protein modification suggested that plants began to adapt to environmental changes and tried to repair a series of injuries caused by low-temperature stress. Furthermore, the redox system also changed rapidly after plants were exposed to low-temperature stress.

In the early stage of cold treatment (8:00–11:00), downregulated genes were enriched only in transport and ribosome synthesis. However, with the extension of time under cold stress, there were varying degrees of enrichment in DNA synthesis and chromatin structure, RNA binding and transcription, amino acid synthesis, protein synthesis and folding, starch synthesis, substance transport and heterologous light system II (Figure 3B; Supplementary Table S3). Based on the enrichment analysis, we identified 18 genes involved in purine synthesis and 6 genes involved in pyrimidine synthesis, which were severely inhibited after low-temperature stress in *Setaria viridis* (Figure 3C). Meanwhile, we identified 29 downregulated genes involved in RNA transcription under low-temperature stress. Twenty-six genes involved in protein folding were severely inhibited by low-temperature stress, indicating the abnormal folding and function of proteins (Figure 3D). Additionally, the genes related to the transport of proteins, especially chloroplast and mitochondrial transport proteins, were severely inhibited by low-temperature stress These results showed that low-temperature stress affected the growth of plants at the levels of DNA, RNA, and protein.

Combined with the upregulated gene enrichment results, we hold that although the plant began to synthesize some of the substances used to resist cold stress after a period of adaptation, in the synthesis of purines and pyrimidines, the structure of chromosomes, RNA transport and translation, protein folding and other basic biochemical processes were severely inhibited. The dynamic transcriptional level may be the main reason for the irreversible damage to *Setaria viridis* at 4 °C.

### 3.3. Changes in Transcription Factors during Chilling Stress

**Figure 4:**
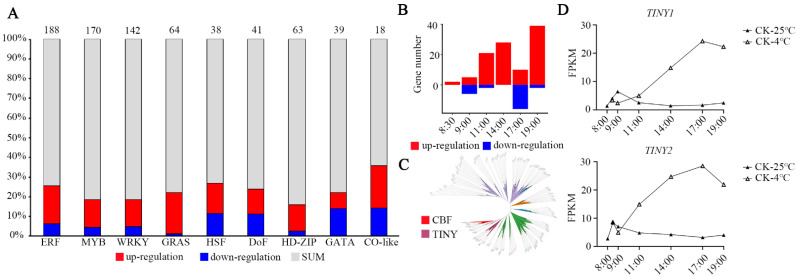
The dynamics of cold-regulated differentially expressed TFs. (**A**) The number of differentially expressed transcription factors after cold treatment. (**B**) The number of differentially expressed genes in ERF family after cold treatment. (**C**) ERF family phylogenetic tree in *Setaria viridis* and *Arabidopsis thaliana*. (**D**) The expression of *TINY1* and *TINY2* at the six time points.

Because transcription factors (TFs) play critical roles in abiotic stress, we analyzed the changes in their expression after low-temperature stress in *Setaria viridis*. A total of 391 differentially expressed TFs were detected. We principally displayed 9 TF families that accounted for 54.47% of all differentially expressed TFs. *AP2/ERF*, *MYB*, *WRKY*, *GRAS*, and *HD-ZIP* were significantly induced by chilling stress (Figure 4A). A MYC-like bHLH transcription factor named ICE1, which is a regulator in the cold-induced transcriptome and freezing tolerance, can be upregulated by CBFs under cold treatment in *Arabidopsis thaliana* [3]. However, the expression of the two identified ICEs was not affected by low temperature in *Setaria viridis* (Supplementary Table S4). CBF1, which belongs to the AP2/ERF TFs, is the key transcription factor involved in cold stress in vascular plants [6]. To identify all genes encoding CBFs, a phylogenetic tree of total AP2/ERF proteins from *Setaria viridis* and *Arabidopsis thaliana* was constructed (Figure 4B,C). Subsequently, we identified that the expression of 1 member from the CBF subfamily and 9 members from the CBF-like subfamily was induced by cold stress (Supplementary Table S4). Unlike the regulation module of *CBF2* in *Arabidopsis*, no genes belonging to the ethylene response factor (ERF) subfamily exhibited negative regulation under cold stress in *Setaria viridis*. Meanwhile, low expression of most *CBFs* was detected before or after cold stress, except for *Sevir.4G016300,* named *CBF-L*, which exhibited drastically increased expression under 4 °C conditions (Supplementary Table S4). This result suggested that *CBF-L* may play an important role in cold stress in *Setaria viridis*. Additionally, 15 members of the TINY subfamily were identified, and 4 genes (*Sevir.1G027700*, *Sevir.1G263700*, *Sevir.7G201200* and *Sevir.9G32900*) had enhanced expression levels after treatment with low temperature (Supplementary Table S4), with their orthologs from *Arabidopsis thaliana* also related to cold, drought, and salt tolerance [44,45]. The expression levels of *TINY1* (*Sevir.7G201200*) and *TINY2* (*Sevir.9G329000*) were strongly induced by low temperature, reaching maximum increases of 15-fold and 9-fold, respectively, relative to the controls at 9 h after 4 °C treatment (Figure 4D). These results suggest that modifying the expression of *CBF-L*, *TINY1*, and *TINY2* may affect cold resistance in *Setaria viridis*. Finally, through basic local alignment search tool P (BLASTP), we identified 81 genes that may be regulated by CBFs according to currently unknown *COR* genes in *Arabidopsis* (Supplementary Table S4) [46].

Transcriptome analysis indicated that only 12% of *COR* genes are directly controlled by CBFs [9]. Therefore, some CBF-independent components must function in cold signaling. For example, the heat stress-responsive transcription factor HSFA1 regulates freezing tolerance in a CBF-independent manner [47]. The *esk1* mutant shows constitutive freezing tolerance that is independent of the CBF regulon in *Arabidopsis* [48]. We found that the *HSFA1* and *ESK1* homologous genes in *Setaria viridis* were also induced by low temperature (Supplementary Table S4). *BBX7* and *BBX8* have recently been reported to be able to enhance freezing tolerance by CRY2-mediated blue-light signaling [31]. We identified 14 genes from the BBX family, 10 of which were induced by cold stress (Supplementary Figure S2; Supplementary Table S4). This result indicates that the BBX family may also play an important role in the response to low-temperature stress in *Setaria viridis.*

### 3.4. Functional Characterization of DEGs under EOD-FR

**Figure 5:**
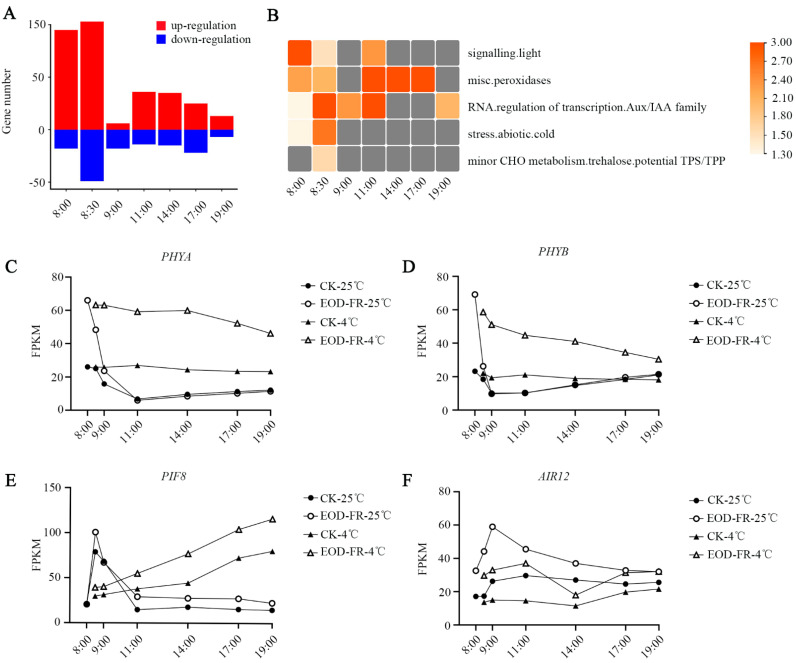
Effects of EOD-FR in *Setaria viridis*. (**A**) The number of differentially expressed genes after EOD-FR treatment. (**B**) MAPMAN enrichment analysis of DEGs after EOD-FR treatment. Only significant categories (FDR < 0.05) were displayed. (**C**–**F**), The expression of *PHYA*(C), *PHYD*(D), *PIF8* (**E**), and *AIR12* (**F**).

To characterize the gene response to EOD-FR in *Setaria viridis*, we compared the expression levels of genes in the EOD-FR samples with the expression levels of genes in the Control-25 °C samples. Based on the criteria of adjusted *p* value < 0.01 and | log_2_ (fold change)| ≥ 1, 324 DEGs were observed in all samples. More than half of the DEGs (68.6%) were upregulated. The number of upregulated DEGs (198) at 8:00 a.m. and 8:30 a.m. accounted for 63.2%, suggesting that EOD-FR may primarily affect gene expression within half an hour after exposure to far-red light conditions (Figure 5A).

Subsequently, we performed MAPMAN enrichment analysis to identify specific biological processes. The upregulated genes were concentrated mainly in light signals, cold stress, TPS metabolism, and peroxidases. (Figure 5B; Supplementary Table S5). The TPS/TPP signal, peroxidase signal and cold signal are closely related to plant stress tolerance, especially cold tolerance [42,49]. Therefore, we believe that the genes induced by EOD-FR may be involved in resisting cold stress in *Setaria viridis*.

In contrast to *Arabidopsis*, only 3 phytochromes were identified in *Setaria viridis*, as well as in maize and rice. The results showed that there were some differences between monocotyledons and dicotyledons in the evolution of phytochromes (Supplementary Figure S3). We found that the expression levels of *PHYA* and *PHYB* were drastically increased under EOD-FR conditions (Figure 5C,D). As reported, *PHYA* has been detected to respond to FR and activate the CBF pathway via JA to enhance cold tolerance in tomato [18]. Interestingly, the expression level of *PHYA* was higher when EOD-FR and cold stress were present simultaneously in *Setaria viridis* (Figure 5C). Therefore, we can infer that EOD-FR could improve the cold tolerance of plants via the PHYA-JA-CBF regulatory pathway. Meanwhile, 14 members of the PIF family were identified (Supplementary Table S4). *PIF8* showed induced expression levels under cold stress or EOD-FR conditions in *Setaria viridis*. Interestingly, a higher expression level of *PIF8* was detected when these two stresses coexisted (Figure 5E). These results suggest that the function of phyA/B and PIF8 in response to light and low temperature signals may be conserved among different species. *HY5* is a positive regulator of light signaling in *Arabidopsis*. However, the expression of two *HY5* genes (*Sevir.4G234900* and *Sevir.1G043300*) was induced by cold stress but did not respond to EOD-FR in *Setaria viridis* (Supplementary Table S4), indicating that different species adopt different ways to respond to far-red light.

In addition to the abovementioned light signal-related genes that have been widely studied, we also found *AIR12*, a gene that could enhance cold tolerance by regulating the content of hydrogen peroxide [50]. Conversely, the expression profile of *AIR12* was decreased under cold conditions in *Setaria viridis*. However, *AIR12* was significantly induced by EOD-FR. If cold and EOD-FR existed simultaneously, the expression level of *AIR12* was similar to the expression level of *AIR12* in the control. The effects of cold and EOD-FR were counterbalanced (Figure 5F), suggesting that far-red light can affect the response to low temperature in *Setaria viridis*. Therefore, we decided to explore the relationship between far-red light signals and low-temperature stress.

As a supplement, we compared the DEGs after cold stress with the DEGs after EOD-FR. A total of 199 genes were shared in both sets of genes (Supplementary Figure S4). These results indicate that EOD-FR may affect cold tolerance in *Setaria viridis*.

### 3.5. Transcriptional Dynamics under EOD-FR and Cold Stress

**Figure 6:**
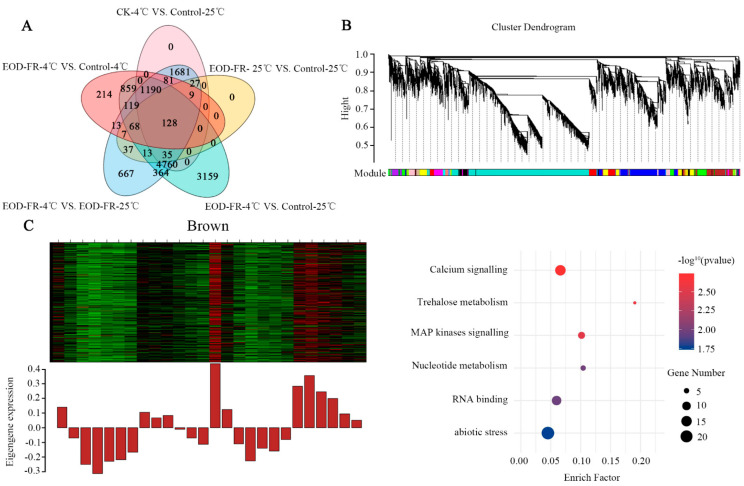
Light–temperature signaling correlation modules. (**A**) Venn diagrams indicating the numbers of upregcontrulated or downregulated genes in four groups. Shared and unique genes in four groups of DEGs. (**B**) Hierarchical cluster tree showing co-expression modules identified by WGCNA. Each leaf in the tree is one gene. The major tree branches constitute 15 modules labeled by different colors. (**C**) Eigengene expression profile and MAPMAN enrichment for the brown module.

To determine the potential genes participating in FR and cold signals, 13431 DEGs were analyzed by WGCNA (Figure 6A). Ultimately, a total of 15 gene network modules were obtained according to pairwise correlation analysis (Figure 6B; Supplementary Figure S5; Supplementary Table S6). We found that the gene expression profiles in the brown module exhibited high expression levels under chilling stress and low expression levels under FR. Then, a higher expression level was detected when cold and EOD-FR coexisted compared with cold conditions (Figure 6C). These results suggest that the genes in these modules may be more sensitive to cold stress. Furthermore, genes related to calcium signaling, trehalose metabolism, and mitogen-activated protein (MAP) kinase signaling were significantly enriched in the brown module (Figure 6C). In addition, genes related to abiotic stress were significantly enriched in the magenta module, midnight blue module, purple module, and turquoise module (Supplementary Table S7).

### 3.6. Identification of TFs Expressed at High Levels in Five Modules

**Figure 7:**
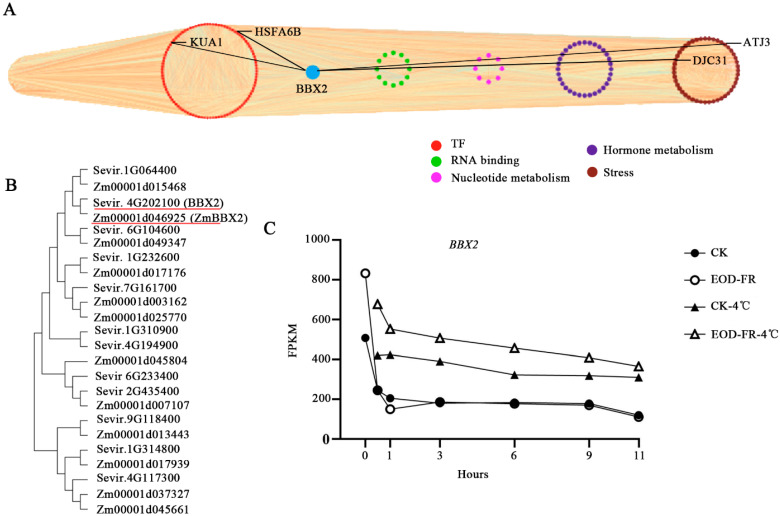
Screening regulated genes from the network hubs in brown module. (**A**) The correlation network of brown module. (**B**) Homology analysis of BBX family in *Setaria viridis* and maize. (**C**) The expression analysis of *BBX2* in response to FR and cold treatment in *Setaria viridis*.

TFs play significant roles in plant growth, development, and various environmental stress adaptations. Thus, TFs in the brown, magenta, midnight blue, purple, and turquoise modules were analyzed, and two TFs belonging to the CO-like family and MYB-like family, *BBX2* and *LHY*, were highly expressed under FR and cold conditions. *BBX2* exhibited high expression levels under chilling stress and low expression levels under FR. Then, a higher expression level of *BBX2* was detected under cold and FR conditions than under cold conditions, but the expression of *LHY* was similar under both cold and cold FR conditions in *Setaria viridis* (Figure 7C; Supplementary Table S4). *BBX7* and *BBX8* were found to be direct HY5 targets that positively regulate freezing tolerance by modulating the expression of a set of cold-responsive genes, which occurs mainly independently of the C-repeat-binding factor pathway in *Arabidopsis thaliana* [31]. Therefore, we suggest that BBX2 may also be a positive regulator of plant cold tolerance. Subsequently, a coexpression network was constructed in the brown module (Figure 7A). The possible gene regulatory pathway of *BBX2* showed that *Sevir.9G055900* (*HSFA6B*), *Sevir.6G064000* (*KUA1*), *Sevir.5G042600* (*DJC31*), and *Sevir.9G154200* (*ATJ3*) may correlate with *BBX2* (Figure 7A). *HSFA6B* responds to abiotic stresses, such as ABA, salinity, and drought, and osmotic stress is individually well established. In addition, *HSFA6a* was found to possibly be able to offer abiotic stress tolerance by regulating reactive oxygen species (ROS) homeostasis in plants [29]. The expression level of *BBX2* was also induced by cold and light in *Arabidopsis thaliana* and maize, implying a similar gene function in different species (Figure 7B) [51,52]. Thus, it is reasonable to assume that this gene expression level may affect resistance to cold and sensitivity to FR. Collectively, this gene may play an important role in the light_temperature signaling pathway in *Setaria viridis*.

## 4. Discussion

The relationship between temperature and light signal has been a hot research topic. Temperature and light signals have been found to share common regulatory elements. The PHYB and PIF3 proteins bind to cold-responsive elements to promote the expression of *COR* genes under low-temperature conditions [30]. However, the genetic basis that transduces the signals between light and temperature to maintain plant growth and adaptation remains elusive. By constructing RNA-Seq data associated with EOD-FR and cold stress, we analyzed changes in transcription levels under cold acclimation and EOD-FR in *Setaria viridis*. The coexpression network, which might be closely related to the light and temperature signals, was constructed through the WGCNA method. The candidate genes we found, such as *LHY* and *BBX2,* may regulate the light or temperature biological pathway (Figure 7).

The enrichment analysis of upregulated genes showed that they participate mainly in the degradation pathway of proteins, lipids, sugars and other compounds at the early stage when plants were exposed to low temperature (Figure 3A). We propose that it is an emergency action to combat cold stress in *Setaria viridis*. On the one hand, this is the reason for the damage to plants caused by low temperature. As an important component of life activity, the degradation of proteins, lipids and carbohydrates will inevitably lead to negative effects. On the other hand, degrading and synthesizing new substances may be a way to cope with stress in plants. For example, starch is an important molecule that mediates plant responses to abiotic stresses, including drought, salinity, and extreme temperature [53].

The downregulated genes were principally involved in DNA chromosomal structure, purine synthesis, RNA binding and transcription, protein folding, and protein and nutrient transport (Figure 3B). The biological processes mentioned above suggest that cold stress could inhibit the vital activity of plant life. The decreased expression of these genes leads directly to plant growth retardation. The low expression of chloroplast-related genes specifically suppresses chlorophyll synthesis, which is one of the main reasons why leaves turn yellow under low-temperature conditions. If the plants are subjected to low temperature for too long, irreversible damage or even death will occur.

TFs play important roles in the regulation of gene expression in response to cold stress, and the variation of expression is proposed as a potential strategy for the genetic improvement of stress tolerance. During the early stage of cold treatment (8:00 a.m. and 9:30 a.m.), the number of DEGs was less than other time points based on the transcriptome dataset. Among these TFs, the members of the ERF occupied the most numbers, indicating that this family may play roles in chilling stress. Thus, genes belonging to ERF were used for further analysis. As in a previous study, CBFs are significant transcription factors in cold tolerance in *Arabidopsis*. In *Setaria viridis*, we also identified 10 CBFs and constructed a phylogenetic tree (Figure 4C and Supplementary Table S4). Interestingly, only one *CBF-L* (*Sevir.4G016300*) was significantly induced by 4 °C, reaching a maximum increase of 400-fold relative to the control at 3 h after 4 °C treatment. The expression levels of the other CBFs were barely detected in seedlings of *Setaria viridis*. In contrast to *Arabidopsis CBF2*, we did not find downregulated CBFs in *Setaria viridis* [8]. There seems to be difference in the cold response network between Arabidopsis and *Setaria viridis*. Taken together, our results provide detailed insight into dynamic changes under cold stress and serve as a valuable resource for functional genomics study in *Setaria viridis*.

After EOD-FR treatment, the DEGs were enriched mainly in the biological pathways of light signal, peroxidase signal, cold stress signal, and TPS/TPP signal, which are closely related to stresses, especially cold stress (Figure 5B). This discovery suggests that the expression of these cold-related genes may be regulated by the EOD-FR signal, thereby promoting the cold tolerance of *Setaria viridis*. Although there were two environmental variables in EOD-FR-4 °C vs. Control-25 °C, the DEGs in this group were enriched in lipid elongation and MGDG synthase, which are closely related to stress resistance.

The DEGs found in different treatments were used to construct a coexpression network and to explore the relationship between the EOD-FR signal and low temperature by WGCNA. The resulting gene network was then analyzed and clustered. A total of 15 informative gene modules that included functions in a wide range of physiological processes were identified. The modules we analyzed are especially useful in that they can assign the probable functions to DEGs and identify participating genes for specific stress-related or physiological processes. According to the increased expression level induced by these two stresses, we focused on the brown, magenta, midnight blue, purple, and turquoise modules for further research. Correspondingly, as revealed by the MAPMAN enrichment analysis, the brown modules included many cold-related genes (Figure 6C). However, the genes in the black modules were not enriched in pathways related to cold stress.

An important feature of our network is that transcription factors and their down-stream genes usually exist in the same module, suggesting similar expression patterns. In the brown module, we found that *BBX2* may regulate or interact with genes, such as *KUA1* and *HSFA6B*. Meanwhile, the expression of *BBX2* reached 3-fold the expression of *BBX2* of the control under low-temperature conditions after acclimation to EOD-FR. Additionally, when cold and FR were present simultaneously, the expression of *BBX2* was higher than the expression of *BBX2* under any one stress (Figure 7A,C). *BBX2*, encoding a CO-like transcription factor, acts as the candidate gene to regulate the crosstalk between FR and cold. From a previous study, BBX proteins play important roles in photomorphogenesis, phytohormones, biotic and abiotic stress responses, shade avoidance, etc. [54,55,56,57,58] For instance, STH3 (a BBX protein) physically interacts with HY5 and COP1 to positively regulate photomorphogenesis in *Arabidopsis* [54]. Another gene named *BBX18* is a heat stress-induced factor that negatively regulates thermotolerance in *Arabidopsis* [58]. Therefore, we propose that *BBX2* may be crucial in sensing EOD-FR and enhancing low-temperature tolerance in plants.

## 5. Conclusions

A large number of independent and interactive genes in response to chilling stress and EOD-FR signaling were identified. Subsequently, a regulatory network was constructed using a well-designed transcriptomics study. *BBX2* was identified as responsive to chilling and EOD-FR through coexpression network analysis. In addition, this dataset will be useful for unravelling the signaling network related to these two environmental factors.

## Data Availability

The datasets used and/or analyzed during the present study are available from the corresponding author on reasonable request.

## Supplementary Materials

The following are available online at https://www.mdpi.com/article/10.3390/genes13091565/s1, Table S1: The DEGs in response to chilling stress. Table S2: Functional category enrichment of upregulated DEGs in response to cold. Table S3: Functional category enrichment of downregulated DEGs in response to cold. Table S4: Changes in expression of genes in response to cold or EOD-FR. Table S5: Functional category enrichment of upregulated DEGs in response to EOD-FR. Table S6: Hierarchical cluster for coexpression modules identified using WGCNA. Table S7: Functional category enrichment of coexpression modules. Figure S1: The number of DEGs under cold stress. Figure S2: BBX family phylogenetic tree in *Setaria viridis* and *Arabidopsis thaliana*. Figure S3: PHY phylogenetic tree in *Setaria viridis*, *Oryza sativa*, *Zea mays* and *Arabidopsis thaliana*. Figure S4: Venn diagram analysis of DEGs under three different treatments. Figure S5: Eigengene expression profile for the 15 modules.
