# Supplementary figures and images for: Transcriptomic Analysis Reveals the Correlation between End-of-Day Far Red Light and Chilling Stress in Setaria viridis
Source: Genes (Basel). 2022 Aug 31;13(9):1565. doi: 10.3390/genes13091565 (PMC9498584; doi:10.3390/genes13091565)

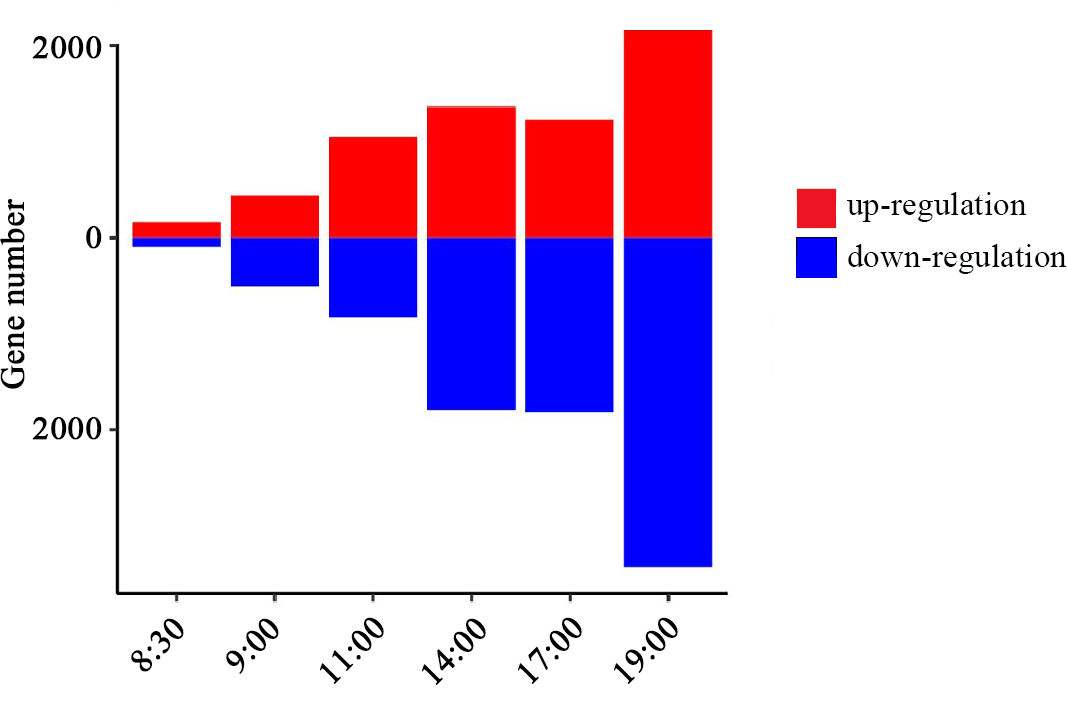

Supplement: Supplementary file 1 [file genes-13-01565-s001.zip › genes-1881213-Figure S1.tif]

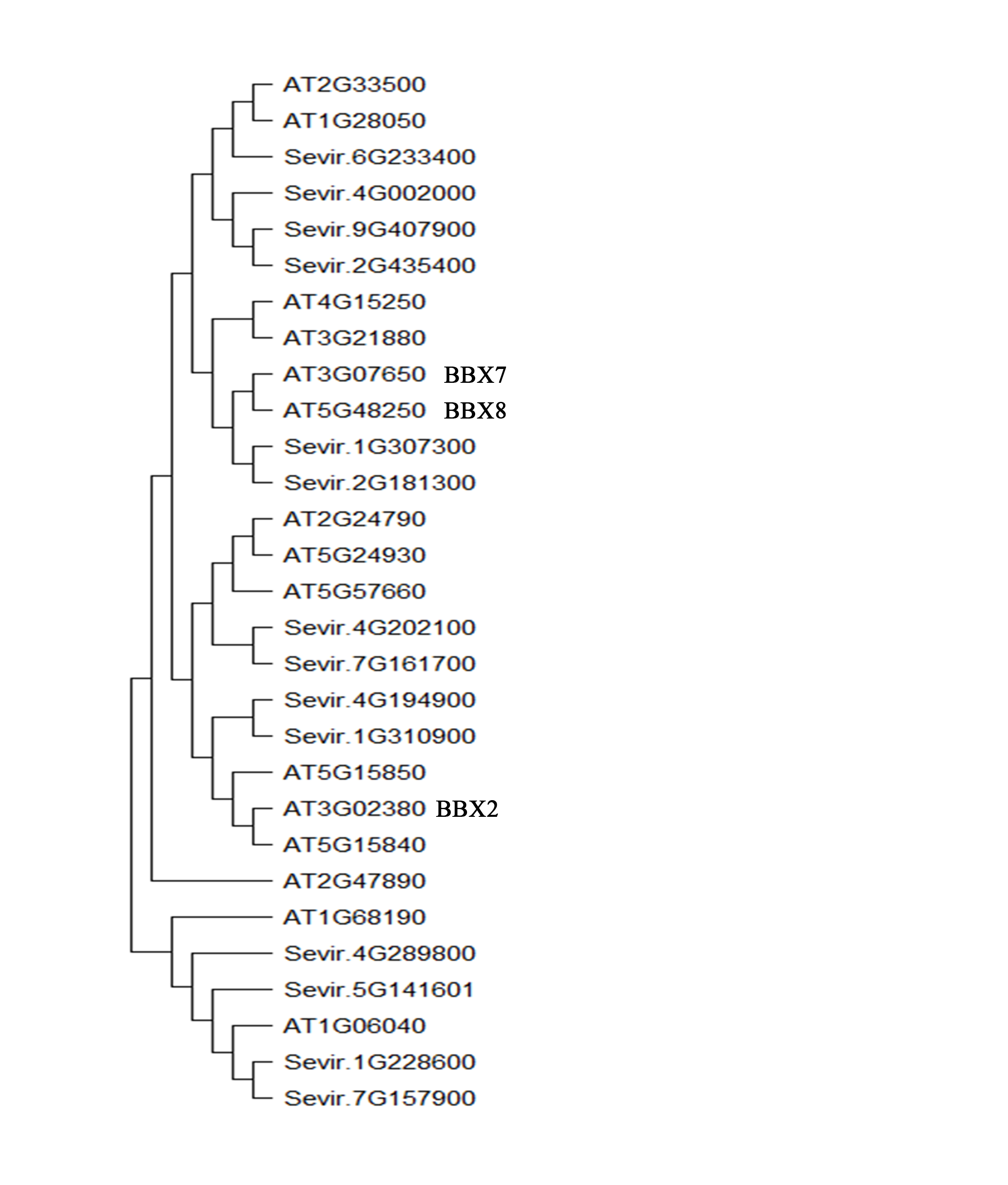

Supplement: Supplementary file 1 [file genes-13-01565-s001.zip › genes-1881213-Figure S2.tif]

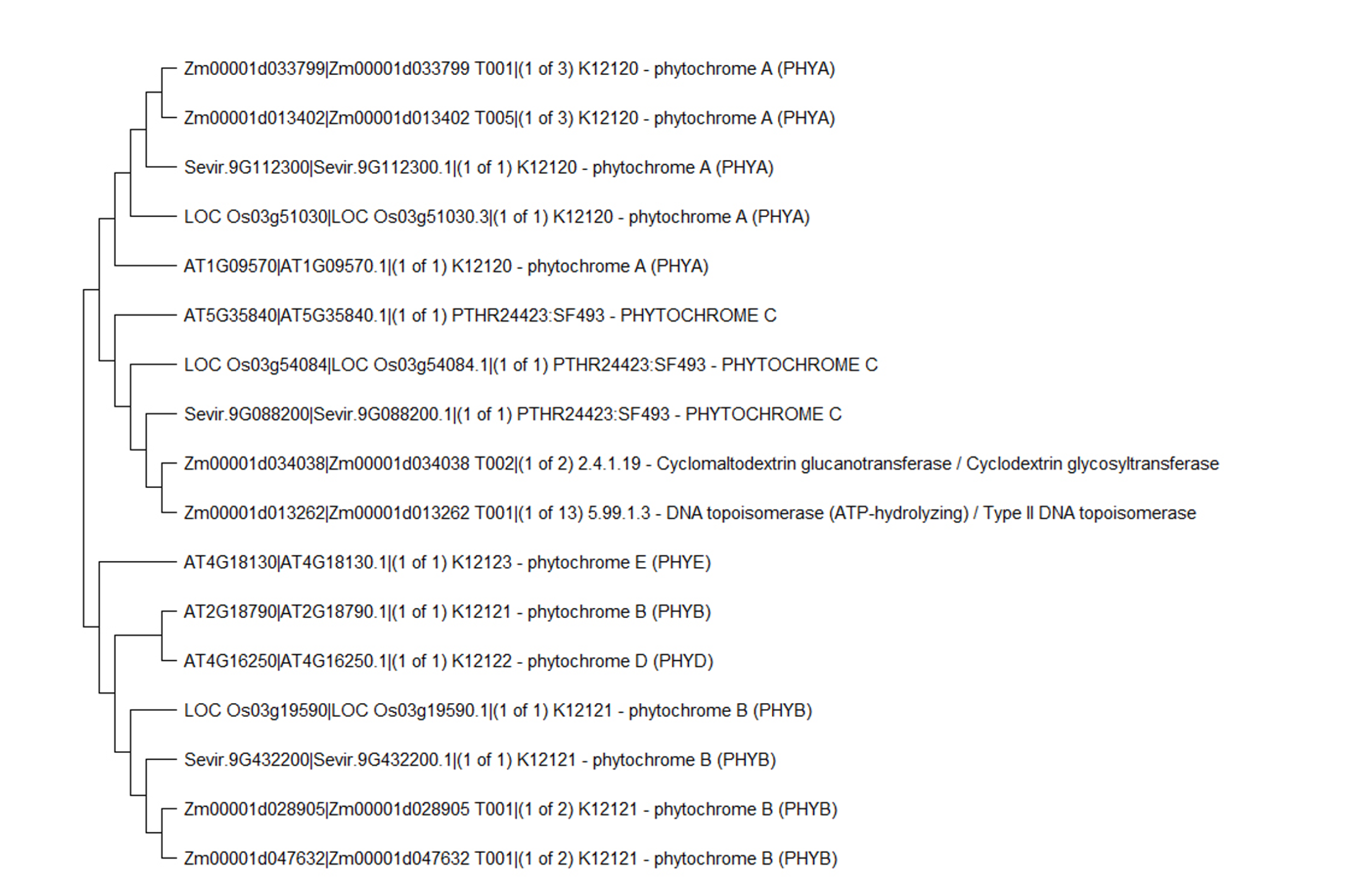

Supplement: Supplementary file 1 [file genes-13-01565-s001.zip › genes-1881213-Figure S3.tif]

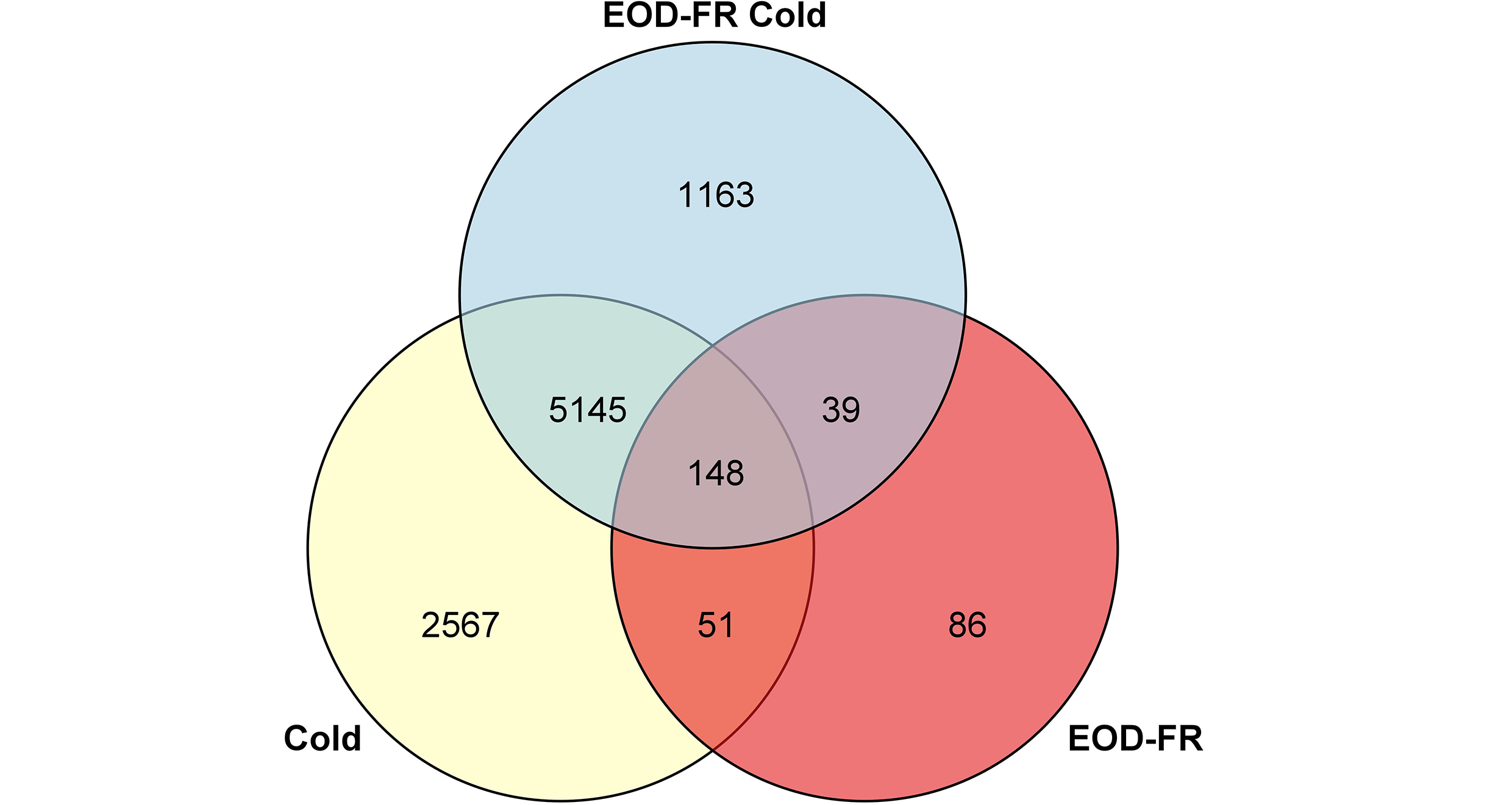

Supplement: Supplementary file 1 [file genes-13-01565-s001.zip › genes-1881213-Figure S4.tif]

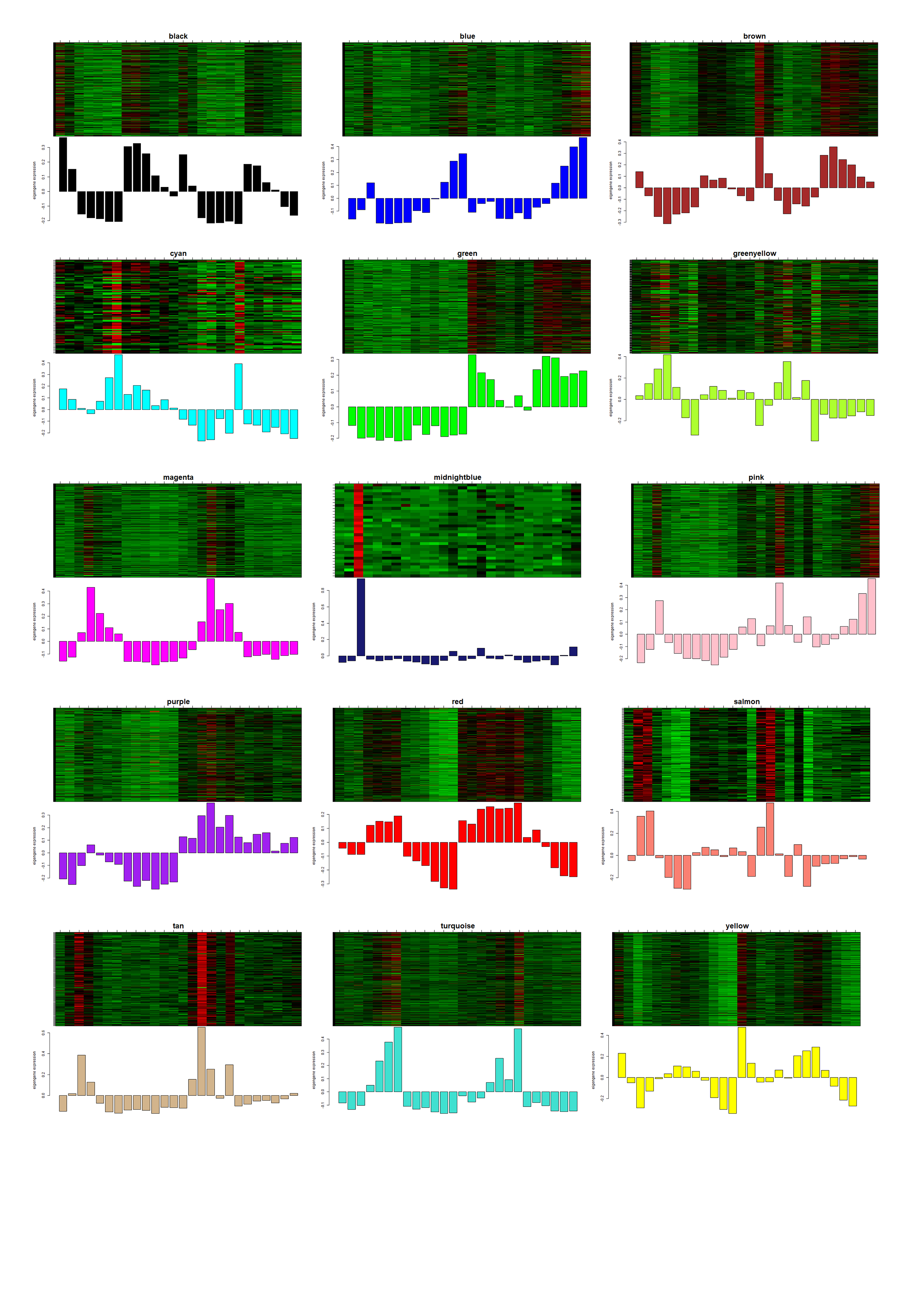

Supplement: Supplementary file 1 [file genes-13-01565-s001.zip › genes-1881213-Figure S5.tif]
